# Enhancing the Photovoltaic Efficiency of In_0.2_Ga_0.8_N/GaN Quantum Well Intermediate Band Solar Cells Using Combined Electric and Magnetic Fields

**DOI:** 10.3390/ma17215219

**Published:** 2024-10-26

**Authors:** Hassan Abboudi, Redouane En-nadir, Mohamed A. Basyooni-M. Kabatas, Ayoub El Baraka, Walid Belaid, Ilyass Ez-zejjari, Haddou El Ghazi, Anouar Jorio, Izeddine Zorkani

**Affiliations:** 1 LPS, Faculty of Sciences, Mohamed Ben Abdellah University, Fes 30000, Moroccoayoub.elbaraka@usmba.ac.ma (A.E.B.);; 2 Department of Precision and Microsystems Engineering, Delft University of Technology, Mekelweg 2, 2628 CD Delft, The Netherlands; 3 School of Physics and Astronomy, Leeds University, Woodhouse Lane, Leeds LS2 9JT, UK; 4 2SMPI Group, ENSAM Laboratory, Hassan II University, Nile 150, Casablanca 20670, Morocco; ezzejjariilyass@gmail.com

**Keywords:** IBSC, photovoltaic, efficiency, III-N materials, electromagnetic fields, parabolic potential

## Abstract

This study presents a theoretical investigation into the photovoltaic efficiency of InGaN/GaN quantum well-based intermediate band solar cells (IBSCs) under the simultaneous influence of electric and magnetic fields. The finite element method is employed to numerically solve the one-dimensional Schrödinger equation within the framework of the effective-mass approximation. Our findings reveal that electric and magnetic fields significantly influence the energy levels of electrons and holes, optical transition energies, open-circuit voltages, short-circuit currents, and overall photovoltaic conversion performances of IBSCs. Furthermore, this research indicates that applying a magnetic field positively influences conversion efficiency. Through the optimization of IBSC parameters, an efficiency of approximately 50% is achievable, surpassing the conventional Shockley–Queisser limit. This theoretical study demonstrates the potential for next-generation photovoltaic technology advancements.

## 1. Introduction

Intermediate band solar cells (IBSCs) are at the forefront of solar cell technology, enhancing efficiency by introducing additional energy levels within the bandgap of single-bandgap solar cells [1]. By facilitating transitions from the valence band (VB) to the intermediate band (IB) and from the IB to the conduction band (CB), IBSCs enable the absorption of sub-bandgap photons, resulting in heightened photocurrents and photovoltages, thus elevating the overall power conversion efficiency (PCE) beyond that of traditional single-bandgap solar cells. This innovation creates intermediate quantized levels within the bandgap, reducing non-radiative doping-related transitions, thereby offering a promising strategy to surpass conventional limits and potentially reduce the cost of photovoltaic electricity production. Achieving optimal performance necessitates ensuring that VB to IB and IB to CB transitions are optically allowed without spectral overlap. Under ideal conditions, IB solar cells boast a theoretical maximum PCE of 63%, a notable improvement over the 41% maximum PCE of single-bandgap solar cells [2]. Since the seminal works of Luque, Marti, Levy, and Nozik, the exploration of the novel generation of solar cells has witnessed significant growth. Overcoming the Shockley–Queisser limit to enhance photovoltaic conversion efficiency (η) remains a formidable challenge for researchers and industry alike [3,4,5]. Theoretically, the electron’s intermediate band was derived using the Kronig–Penney model to solve the Schrödinger equation. These findings support enhancing the photoelectric conversion efficiency by adjusting the indium concentration, the well and/or dot size, and inter-spacing [6]. IBSCs exploit their inherent intermediate band by exploiting nanostructured semiconductor materials, such as quantum wells (QWs) and quantum dots (QDs). Quantum confinement effects, resulting from the small size of QWs or QDs, promote the formation of this intermediate band, requiring a high QD concentration for effective band formation [7]. Manipulating these nanostructures’ properties, such as size, shape, morphology, and inter-dot separation, offers avenues for further enhancing IBSC PCE [8].

Recently, an experiment demonstrated that materials utilized in IBSCs include (In, Ga) self-assembly in the GaAs matrix, InAs QDs in the GaAs matrix, and GaSb QDs in the GaAs matrix, showcasing promising PCEs of up to 18% [9]. Moreover, nanostructures, particularly quantum wells, play a pivotal role in enhancing the performances of photovoltaic cells. Rooted in III-N semiconductors, these nanostructures offer distinct advantages over conventional semiconductors, such as heightened energy efficiency and durability. In addition, the InGaN ternary alloy demonstrates the remarkable ability to absorb the entire visible spectrum, spanning from infrared (IR) to ultraviolet (UV) wavelengths, contingent upon variations in indium concentration. Nonetheless, achieving comprehensive spectral absorption poses challenges during growth, primarily attributable to these materials’ localized and polar nature, particularly when indium concentrations exceed 50% [10].

In the realm of photovoltaic conversion efficiency concerning intermediate band solar cells based on quantum dots and quantum wells, numerous studies have been documented in the literature. El Aouami et al. [11] conducted a theoretical investigation into the photovoltaic conversion efficiency of InGaN/GaN solar cells, focusing on integrating a supra-crystal array of QDs within the i-region of a photodiode. Imran et al. [12] reported increased efficiency achieved via a planar intermediate band in QD-based InAs/GaAs solar cells. Recently, El Ghazi et al. [13] used the finite element method to study key characteristics of InGaN/GaN intermediate band solar cells (IBSCs), focusing on X-sun concentration effects. The same research group examined the operational mechanisms of n-In_0.42_Ga_0.58_N/i-(In, Ga)N/p-In_0.42_Ga_0.58_N solar cells at room temperature using experimental data from the AM1.5D, AM1.5G, and AM0 American Society for Testing and Materials datasets [14]. Furthermore, over the past two years, our research has delved into the temperature-dependent photovoltaic properties of (In, Ga)N single-intermediate band quantum well solar cells, exploring various geometries [15]. Additionally, we examined the impurity-induced photovoltaic efficiency of (In, Ga)N/GaN quantum well single single-intermediate band solar cells, focusing on heavy-hole impact [16]. Most recently, our investigations have centered on the efficiency of InN/InGaN/GaN intermediate band solar cells, considering the influences of hydrostatic pressure, In-compositions, built-in electric fields, confinement, and thickness [17].

This study represents the culmination of our recent endeavors. It presents a groundbreaking approach: integrating electric and magnetic fields to enhance the characteristics of InGaN/GaN-based intermediate band solar cells (IBSCs). Remarkably, these factors have been overlooked in prior research despite their potential to yield substantial improvements in efficiency and stability. This innovative strategy offers promising opportunities for optimizing IBSCs, emphasizing the critical importance of incorporating these considerations into the design of next-generation, high-performance solar cells.

## 2. Theory and Models

**Figure 1:**
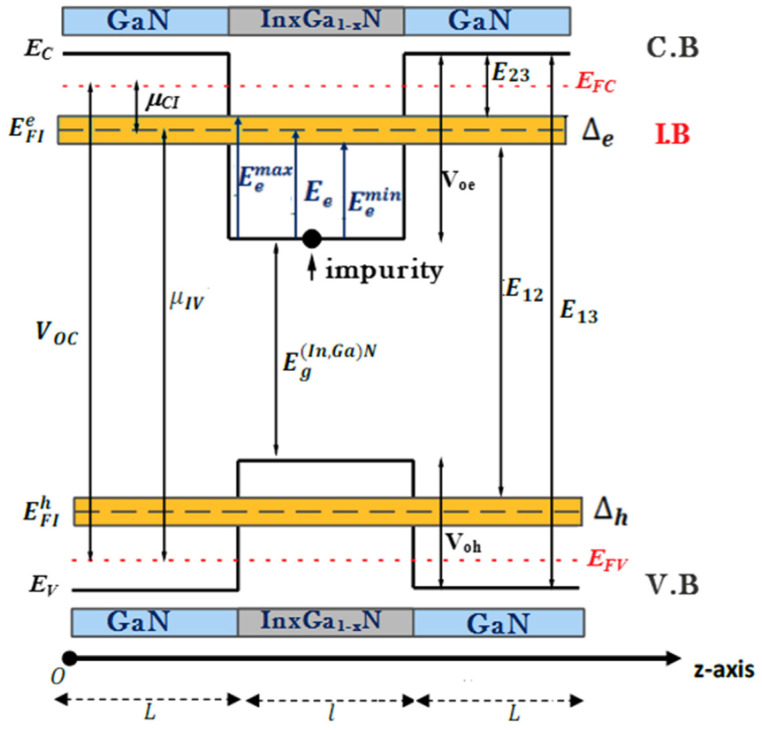
A schematic diagram of a GaN/(In,Ga)N/GaN QW single-intermediate band implanted in the conventional solar cell under study.

The depicted system comprised a p-GaN/(In,Ga)N/GaN-n solar cell, as illustrated in Figure 1. It featured a GaN layer of thickness L serving as a barrier and an intrinsic (In,Ga)N layer of thickness l acting as the well width. The investigation excluded considerations of n(p)-type doping effects through the Poisson equation. This configuration represented a quantum well intermediate band solar cell (QW-IBSC) model, as delineated in Figure 1, depicting the schematic of the designed intermediate band solar cell (IBSC). The Schrödinger equation governing the energy levels of ground and excited states within a parabolic quantum well (PQW), subject to a parabolic confinement potential, was solved using the finite element method (FEM). The use of the FEM in our study for solving the Schrödinger equation within the effective-mass approximation had inherent limitations. Specifically, this approach did not account for complex interactions such as self-polarization or multi-particle effects, which are crucial for accurately modeling quantum well intermediate band solar cells (QW-IBSCs). Recognizing these limitations, we acknowledge that our findings represent a foundational analysis, and we encourage future research to incorporate more sophisticated models to enhance the realism and applicability of the results.

This numerical technique was employed due to the system’s intricate nature, particularly when incorporating a hydrogen-like impurity at the system’s center, specifically at L+l/2.

In Figure 1, $E_{C}$, $E_{V}$, $E_{FC}$, $E_{FV}$, and $E_{FI}$, respectively, denote the energies of the conduction and valence bands and their quasi-Fermi levels. Additionally, $E_{FI}$ Represent the quasi-Fermi level energy of the IB. CB and VB represent the conduction and valence bands, respectively. $\mu_{CI}$ and $\mu_{IV}$ denote the chemical potentials between the CB, VB, and the IB, while $\mu_{CV}$ represents the chemical potential between the CB and VB. $E_{23}$ and $E_{12}$ symbolize the bottom and top sub-band gap energies, respectively.

**Figure 2:**
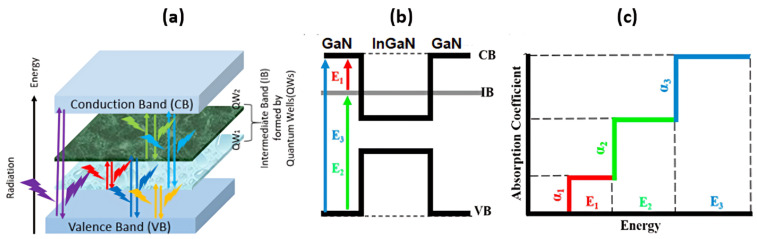
(**a**) The visualization of radiative potential optical transitions within an intermediate band (IB). (**b**) A diagram depicting the band structure of the intermediate band formed by quantum wells (QWs). (**c**) An explanation of the three-step absorption process’ formation.

Figure 2 illustrates the enhanced absorption capability of the proposed structure compared to conventional p-n structures. The figure comprises three parts: (a) a visualization of radiative potential optical transitions within an intermediate band (IB), (b) a diagram illustrating the band structure of the intermediate band formed by quantum wells (QWs), and (c) an explanation of the three-step absorption process’ formation. The visualization in part (a) depicts the radiative potential optical transitions within the intermediate band (IB). This highlights the ability of the IB to facilitate multiple photon absorptions through radiative transitions, indicating its potential for efficient light harvesting. Part (b) presents a diagram illustrating the band structure of the intermediate band formed by quantum wells (QWs). This depiction showcases the energy levels within the IB and its relationship with the surrounding band structure, providing insights into how the IB enables enhanced absorption capabilities. Part (c) explains the formation of the three-step absorption process. This likely describes how the IB structure allows for the absorption of three photons in two steps, leading to significantly higher absorption than conventional structures. This explanation elucidates the mechanism behind the enhanced absorption observed in the proposed structure, further supporting its potential for improved solar energy conversion efficiency.

### 2.1. Electronic Characteristics

In this work, we considered an electron (hole) confined in a parabolic potential, with the donor impurity placed at the center of the well under the simultaneous influence of electric and magnetic fields. The electric field was applied parallel to the growth direction (z-direction), and the magnetic field was applied perpendicular to the growth direction or electric field. Considering the effective-mass approximation, the energy levels and associated eigenwave functions within this system were determined by solving the time-independent Schrödinger equation:

(1) $$H\Psi \left(z\right)=E\Psi (z)$$

We defined the z-axis to be along the growth axis and applied the electric field in the growth direction and the magnetic field to be used in the x-axis, i.e., $\vec{B}$ = (B, 0, 0). We chose a vector potential $\vec{A}$ in the form $\vec{A}$ = (0, $-B_{z}$, 0) to describe the applied magnetic field.

The gauge chosen to describe the magnetic field effect implied that the magnetic vector potential had to satisfy the following two conditions:

$$(i)\vec{\nabla }\cdot \vec{A}=0$$

$$\left(ii)\vec{A}=\frac{1}{2}\vec{(r}\times \vec{B}\right)$$

The condition (i), the extensive Hamiltonian in Equation (1), which includes particle kinetic energy, confinement potential energy, the impact of impurities, and electromagnetic excitation, is expressed as

(2) $$H=\frac{1}{2m_{i}^{*}}{\left[p+\frac{e}{c}A\left(r\right)\right]}^{2}+V_{0j}^{i}\left(x,y,z\right)\pm \frac{\alpha e^{2}}{\varepsilon_{r}^{*}\left|{\vec{r}}_{i}-{\vec{r}}_{0}\right|}\pm q\vec{F}\cdot \vec{r}\;\left(i=e,h\right)$$

We noticed that the signs (−) and (+) correspond to the electron and hole, where $m_{i}^{*}$ is the electron (hole) effective mass, and e is the electron charge. p is the momentum, while |$\vec{r_{i}}$ − $\vec{r_{0}}$| is the particle impurity distance. Here, α = 0 when there is no impurity center and α = 1 when the impurity has been considered. In $A(r)=\frac{1}{{2m}_{i}^{*}}$ (B×r), showing the vector potential of magnetic field B, $\varepsilon_{r}^{*}$ is the relative dielectric constant of different materials in this study, and $V_{0j}^{i}(x,y,z)$ is the confinement potential profile for the electron (hole) in the z-direction. Within the framework of one-band parabolic effective-mass theory, the (In,Ga)N/GaN sub-bands structure was obtained by solving the impurity-related time-independent Schrödinger equation in the presence of magnetic and electric field; the equation is expressed as [18]:

(3) $$-\frac{\hbar^{2}}{2m_{i}^{*}}\frac{\partial^{2}\psi_{i}\left(z\right)}{\partial z^{2}}+\left(V_{0j}^{i}\left(z\right)\pm e\mu \cdot z-\frac{\alpha e^{2}}{\varepsilon_{0}\varepsilon^{*}\left(x\right)\sqrt{{\left(z-z_{0}\right)}^{2}+2}}+\frac{e^{2}\gamma^{2}z^{2}}{2m_{i}^{*}c^{2}}\right)\psi_{i}\left(z\right)={E_{i}\psi }_{i}\left(z\right)$$

Notice that we restricted ourselves to the case corresponding to the neglect of the self-polarization potential of particles (electrons and holes) due to the interaction of the particle and its image charge, as described in reference [19]. From this perspective, taking into account the conduction and valence discontinuities, the finite particle confinement potential was assumed to be parabolic in shape and expressed as follows:

(4) $$V_{0j}^{i}\left(z\right)=\left\{\begin{array}{cc} \frac{4Q^{i}∆E_{g}\left(x\right)}{l^{2}}{\left(z_{i}-L-\frac{l}{2}\right)}^{2} & \mathrm{for}\;L\leq z_{i}\leq L+l\\ Q^{i}∆E_{g}\left(x\right) & elsewhere \end{array}\right.$$

where $Q^{i}=0.7(0.3)$ is the conduction (valence) band offset, and $∆E_{g}\left(x\right)$ is the band-gap energy difference between GaN and ${In}_{x}Ga_{1-x}N$ governed by the In-fraction given as [13]

(5) $$∆E_{g}\left(x\right)=E_{g}^{GaN}-E_{g}^{{In}_{x}Ga_{1-x}N}\left(x\right)$$

The ${In}_{x}Ga_{1-x}N$ band gap energy is expressed using the following quadratic function of the In-concentration:

(6) $$E_{g}^{{In}_{x}Ga_{1-x}N}\left(x\right)=x\cdot E_{g}^{InN}+{\left(1-x\right)\cdot E}_{g}^{GaN}-Cx\left(1-x\right)$$

where $C\left(=1.43\;eV\right)$ is the band gap bowing parameter. $E_{g}^{InN}$ and $E_{g}^{GaN}$ are the band gap energies of InN and GaN, respectively.

The effective mass and the relative dielectric constant in the different regions are expressed as follows [20,21,22]:

(7) $$m_{i}^{*}(x)=\left\{\begin{array}{cc} x\cdot m_{i,InN}^{*}+{\left(1-x\right)m}_{i,GaN}^{*} & L<Z\leq L+l\\ m_{i,GaN}^{*} & elsewhere \end{array}\right.$$

(8) $$\varepsilon_{r}^{*}(x)=\left\{\begin{array}{cc} x\cdot {\varepsilon^{*}}_{InN}+\left(1-x\right)\cdot {\varepsilon^{*}}_{GaN} & L\leq Z\leq L+l\\ {\varepsilon^{*}}_{GaN} & elsewhere \end{array}\right.$$

Given that an analytical solution to such a nonlinear Schrödinger equation is impossible, the finite element method (FEM) was used to solve it using a mono-directional mesh (calculation grid) consisting of 3N + 1 points. We used this numerical method to numerically solve the Schrödinger equation for our quantum system because of its accuracy, which is affected by various factors such as mesh size, basic function order, and element type. It accurately calculates the ground and low-lying excited states of simple quantum systems like the harmonic oscillator or the particle in a box. For more complex systems or higher excited states, the accuracy of the FEM solution may decrease due to the need. Notice that to obtain the energy levels and their corresponding wave functions, the z-axis 1D-Schrödinger equation ($y-y_{0}=x-x_{0}=1$) was numerically solved considering the following boundary conditions:

(9) $${\left[\underset{n}{\to }\cdot \underset{\nabla }{\to }\left(\frac{\psi }{m_{e,b}^{*}}\right)\right]}_{b}={\left[\underset{n}{\to }\cdot \underset{\nabla }{\to }\left(\frac{\psi }{m_{e,w}^{*}}\right)\right]}_{w}$$

The studied system makes use of a mesh grid with 3N + 1 points. Each layer is discretized using various discretization steps. The barrier step is $h_{b}=L/N$, whereas the well region step is $h_{w}=l/N$. For 0<k<N, the mesh nodes of a single QW are given as follows: the left barrier is $z_{k}=k*h_{b}$, the well region is $z_{k}=L+k*h_{w}$, and the right barrier is $z_{k}=L+l+k*h_{b}$. The first and second derivative wave functions are as follows:

(10) $${\left.\frac{\partial^{2}\psi (z)}{\partial z^{2}}\right)}_{z_{k}}=\frac{\psi_{k+1}-2\psi_{k}+\psi_{k-1}}{{\left(z_{k+1}-z_{k}\right)}^{2}}$$

(11) $${\left.\frac{\partial \psi \left(z\right)}{\partial z}\right)}_{z_{k}}=\frac{\psi_{k+1}-\psi_{k}}{z_{k+1}-z_{k}}$$

### 2.2. Photonic Characteristics Related to Optical Transitions in IBSCs

Achieving broad absorption across the entire light spectrum is crucial to improve the efficiency of photoelectric conversion. Within this framework, three distinct optical transitions emerged: from the valence band (VB) to the intermediate band (IB), from the IB to the conduction band (CB), and directly from the VB to the CB. In conventional solar cells, electron-hole pairs are typically generated only by photons possessing energies surpassing the band gap energy. Nevertheless, within intermediate band solar cell (IBSC) systems, photons possessing energies below the band gap energy can also be absorbed through both the initial and secondary optical transitions [23]. Certain assumptions become imperative considering the prerequisites delineated above for the effective operation of intermediate band solar cells (IBSCs). These encompass ensuring the electrical isolation of the IB layer from external contacts, refraining from extracting current from the IB layer, and mandating that all transitions between the valence band (VB), the intermediate band (IB), and the conduction band (CB) be radiative, maintaining constant quasi-Fermi levels corresponding to each band, and ensuring adequate device thickness for thorough absorption. In addition, the parameters of utmost interest for solar cells are photocurrent density, open circuit voltage, and efficiency. Additionally, under full-concentration sunlight conditions, the photo-generated density of the solar cell is determined by the number of absorbed and emitted photons. Referring to the energy band diagram of the IBSC depicted in Figure 1, the photocurrent density ($j_{sc}$) can be expressed according to the methodology outlined in ref. [24]:

(12) $$\frac{j_{sc}}{e}=\left[F\left(E_{13},\infty ,T_{s},0\right)-F\left(E_{13},\infty ,T_{c},\mu_{CV}\right)\right]+\left[F\left(E_{23},E_{12},T_{s},0\right)-F\left(E_{23},E_{12},T_{c},\mu_{CI}\right)\right]$$

where $T_{s}$ and $T_{c}$ denote the surface temperatures of the sun and the solar cell, respectively. $\mu_{CV}$ represents the chemical potential between the conduction band (CB) and the valence band (VB), while $\mu_{CI}$ represents the chemical potential between the intermediate band (IB) and the CB. It is notable that all transition energies ($E_{13}$, $E_{12}$, and $E_{23}$) are explicitly delineated in Figure 1. Furthermore, following the Roosbroeck–Shockley formula, the flux F of photons emanating from an object at temperature T can be expressed as per the formula in references [25,26]:

(13) $$F\left(E_{u},E_{v},T,\mu \right)=\frac{2\pi }{h^{3}c^{2}}\int_{E_{u}}^{E_{v}}\frac{E^{2}dE}{e^{\frac{\left(E-\mu \right)}{k_{B}T}}-1}$$

where $E_{u}$ and $E_{v}$ are the lower and upper energy limits of the photon flux for the corresponding transitions, T is the temperature, h is Plank’s constant, c is the light speed in a vacuum, $K_{B}(\approx 1.38\times 10^{-23}\;J/K)$ is Boltzmann’s constant, and μ is the chemical potential of the transition.

On the other hand, the IBSC output voltage $V_{oc}$ for a p-i-n solar cell can be expressed as follows:

(14) $$V_{oc}=\mu_{CV}=\mu_{CI}+\mu_{IV}$$

where $\mu_{CI}$ and $\mu_{IV}$ are given in [26] as follows:

(15) $$\left\{\begin{array}{c} \mu_{CI}=E_{23}+0.5∆e-E_{c}+E_{FC}\\ \mu_{IV}=E_{12}+0.5∆e-E_{FV}+E_{V}+V_{0h}+E_{h}^{1} \end{array}\right.$$

The electron IB width is ∆e.

The quasi-Fermi levels, denoted as $E_{FC}$ for the conduction band (CB) and $E_{FV}$ for the valence band (VB), can be mathematically represented as shown in [15]:

(16) $$\left\{\begin{array}{c} E_{c}-E_{Fc}=kT\;\ln\left(\frac{N_{c}}{n}\right)\\ E_{FV}-E_{V}=kT\;\ln\left(\frac{N_{V}}{p}\right) \end{array}\right.$$

$N_{C}$ and $N_{V}$ represent the effective densities of the conduction band (CB) and valence band (VB), respectively, while n and p denote the concentrations of electrons and holes, as shown in [27]:

(17) $$\left\{\begin{array}{c} n=N_{c}\;exp\left[-\frac{Q∆E_{g}\left(x,T\right)}{K_{B}T}\right]\\ p=N_{V}\;exp\left[-\frac{(1-Q)∆E_{g}\left(x,T\right)}{K_{B}T}\right] \end{array}\right.$$

For greater realism, we employed a fill factor (FF) expression dependent on the open-circuit voltage, as shown below [27]: $V_{th}$ = $\frac{KT}{q}$

(18) $$FF=\frac{\frac{V_{oc}}{V_{th}}-ln\left(\frac{V_{oc}}{V_{th}}+0.72\right)}{1+\frac{V_{oc}}{V_{th}}}$$

Hence, the photovoltaic conversion efficiency could be derived from the output voltage and photocurrent density, as described in [14,28]:

(19) $$\eta =\frac{V_{oc}\cdot J_{sc}\cdot FF}{P_{in}}$$

$P_{in}$ represents the incident solar power per unit area per the Stefan–Boltzmann law.

(20) $$P_{in}=\sigma T_{s}^{4}$$

where $\sigma =5.67\times 10^{-8}\;Wm^{-2}K^{-4}$.

## 3. Results and Discussion

In the present study, the key objective is to investigate the performances of GaN/${In}_{x}{Ga}_{1-x}N$/GaN QW-IBSCs under the combined influence of electric and magnetic fields and impurities, considering the involvement of heavy holes. All physical parameters employed in the numerical calculations are listed in Table 1. Throughout this study, we conducted experiments at room temperature (T = 300 K) and adopted a structure dimension of $L=2\times l=4a^{*}$. To simplify our calculations, we used effective units. $R_{b}^{*}$= $\frac{m_{i}^{*}e^{4}}{2{\left(4\pi \varepsilon^{*}\varepsilon_{0}\right)}^{2}\hbar^{2}}=(29.12\;meV)$ was used as the unit of the energy, and the effective Bohr radius $a^{*}$ = $\frac{{{4\pi \varepsilon }_{0}\varepsilon }^{*}\hbar^{2}}{m_{i}^{*}e^{2}}=(2.53\;nm)$ was used as the unit of length. F = $\frac{ea^{*}\mu }{R_{b}^{*}}$ was used as a dimensionless parameter taking into account the electric field effect, while $B=\frac{e^{2}\gamma^{2}a^{*}}{{2m}_{i}^{*}c^{2}R^{*}}$ was used as a dimensionless parameter depending on the applied magnetic field. For F = B = 1, μ=6.13 kV/cm, and γ=0.19 T, to determine the precise values of F and B used in the interpretation section, we multiplied the values of F and B by the constants μ and γ, respectively.

**Figure 3:**
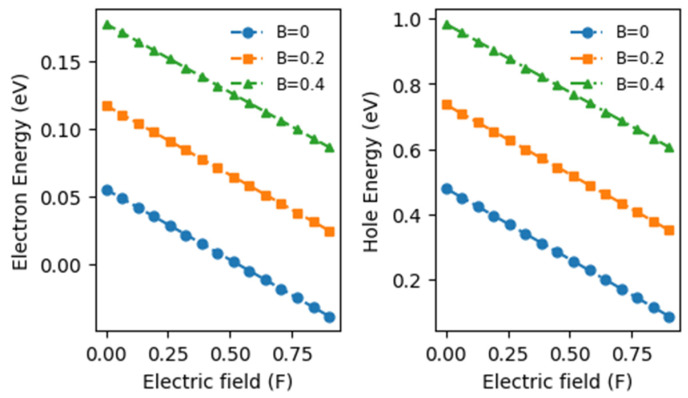
Variations in electron and hole energy within the conduction band (CB) and valence band (VB) as a function of the effective electric field intensity for three specified values of the applied magnetic field.

Figure 3 illustrates the changes in electron (Panel (left) and hole energies (Panel (right)) concerning variations in the electric field, considering three different magnetic field intensities. A notable observation emerges: electron and hole energies exhibit a linear decrease with increasing electric field intensity, regardless of the magnetic field strength. Specifically, for B = 0.4 T, the electron energy reduces from 0.177 eV to 0.098 eV, respectively, and the hole energy reduces from 0.983 eV to 0.648 eV, respectively, as the electric field intensity (F) rises from 0 to 0.8. This signifies decreases of 180.61% and 151.70% for the electron and hole energies, respectively, resulting in an energy shift towards the red spectrum. In addition, this red shift occurs due to the weakening Coulomb interaction between the electron and a donor impurity at the well’s center as the electric field strengthens. Moreover, electron and heavy hole energies increase with rising magnetic field intensity at a constant electric field intensity. For instance, at F = 0.4, the electron energy ($E_{e}$) shifts from 0.0145 eV to 0.1365 eV as B increases from 0 to 0.4, marking a 941.38% increase.

This leads to an energy shift towards the blue spectrum. Consequently, these shifts in energy result from significant modifications to the quantum well’s electrical states under the combined influence of crossed electric and magnetic fields. The confinement potential and the potential arising from the applied fields contribute to these modifications. Additionally, as the magnetic field strength grows, the electrical state levels approach each other, causing an increase in binding energy. This increase stems from enhanced electron confinement due to energy level quantization under the influence of the magnetic field.

**Figure 4:**
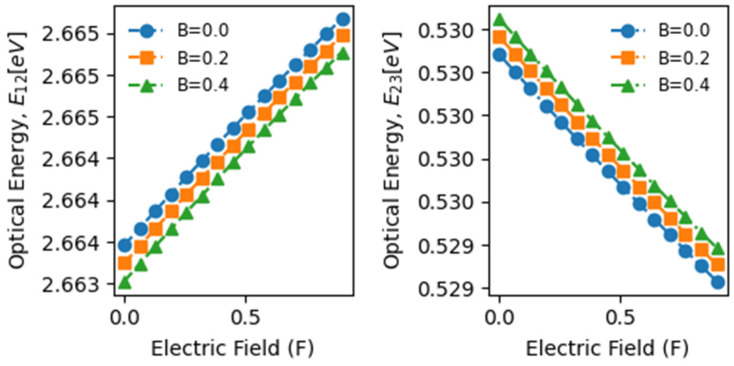
Variations in the optical transition energy between electron and hole levels as a function of the effective electric field intensity for the three specified values of the applied effective magnetic field.

Figure 4 displays the optical transition energies plotted against the electric field (F) for three magnetic field values. Expectedly, $E_{13}$, representing the bandgap of GaN, remains unaffected by these variations. Since $E_{e}$ and $E_{h}$ are strongly influenced by electric and magnetic fields, it is logical to anticipate a similar dependence in optical transition energies. The electric field impacts $E_{e}$ and $E_{h}$, causing decreases in their values. According to our calculations, $E_{23}$ decreases with an increasing electric field regardless of the magnetic field strength, while $E_{12}$ exhibits a consistent increase. Specifically, the primary optical transition energy, $E_{12}$, rises from 2.6635 eV to 2.6650 eV, indicating a 1.5 meV increase, i.e., nearly a 0.6% rise, resulting in a slight blue shift, indicative of the enhanced absorption of low-energy photons.

Conversely, $E_{23}$ decreases from 0.5298 eV to 0.5294 eV, reflecting a 0.4 meV decrease, i.e., a drop of 0.08%, indicating a very slight red shift. The impact of the magnetic field on the changes in the optical transition energies $E_{12}$ and $E_{23}$ is minimal across the specified magnetic field range (0–0.4). Moreover, for a fixed electric field value, the optical transition energy $E_{12}$ is slightly higher in the absence of a magnetic field than when one is present. Conversely, the optical transition energy $E_{23}$ experiences a slight increase as the magnetic field varies from 0 to 0.4, contributing slightly to enhanced absorption and photo-generated current density.

To explore electron and heavy-hole ground-state energies and optical transitions within our structure, we now turn our attention to two key parameters influencing photovoltaic conversion: the open-circuit voltage ($V_{oc}$) and the density of the photo-generated current ($J_{sc}$). We focused on crossed electric and magnetic fields, specifically examining an electric field parallel to the growth axis (z-axis) and a magnetic field perpendicular to it.

**Figure 5:**
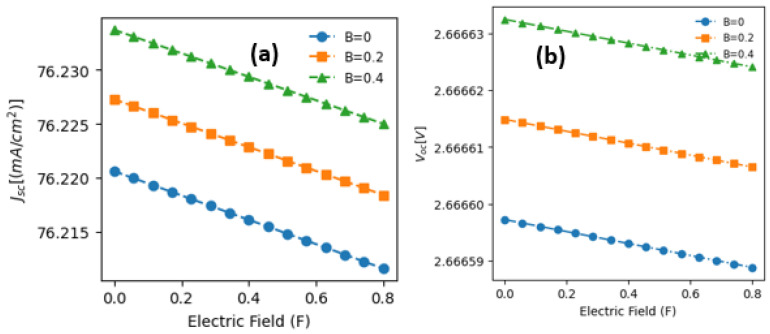
Variations in (**a**) the short-circuit current ($J_{sc}$) and (**b**) the open-circuit voltage ($V_{oc}$) as a function of electric field intensity for three specified values of the applied magnetic field.

Figure 5a depicts the variations in the open-circuit voltage in the QW-IBSC concerning the electric field for three different magnetic field values. Notably, conducting calculations at room temperature with an impurity positioned at the center of the well, discernible trends in the behavior of ($V_{oc}$) emerge. We observe a gradual and marginal decline in ($V_{oc}$) as the electric field strength increases, regardless of the magnetic field’s influence. Specifically, as the electric field intensity ranges from 0 to 0.8, ($V_{oc}$) shifts from 2.66663 V to 2.66662 V with a constant magnetic field of 0.4. This stability indicates a nearly constant voltage despite electric field variations.

Moreover, with an escalation in the magnetic field strength, ($V_{oc}$) demonstrates a modest linear increase. For example, at an electric field strength of 0.4, ($V_{oc}$) changes slightly from 2.66659 V to 2.66663 V, representing a minor rise of $1.5\times 10^{3}\%$ within the analyzed magnetic field range. This phenomenon arises from the accumulation of charge carriers near the junction induced by the heightened magnetic field, leading to a slight augmentation in ($V_{oc}$). Overall, within the studied ranges of electric and magnetic fields, the variations in ($V_{oc}$) are exceedingly slight, underscoring the importance of the obtained data and highlighting the beneficial impacts of electric and magnetic fields on the open-circuit voltage. Figure 5b demonstrates the variations in the density of the photo-generated current, $j_{sc}$, influenced by the electric field for three distinct magnetic field strengths. Notably, the highest densities of photo-generated current consistently occur under zero electric field conditions, regardless of the magnetic field intensity. As the electric field strength increases, there is a gradual and linear decline in the photo-generated current density, irrespective of the magnetic field’s magnitude. Two primary factors can explain this phenomenon: firstly, the asymmetrical restructuring of the quantum well under a non-zero electric field (F≠0), and secondly, the shifting of electron and hole energy levels away from their optimal positions with increasing electric field, thereby impacting optical transitions. $E_{12}$ and $E_{23}$ consequently reduce photon absorption and the number of charge carriers participating in the photo-generated current density. The marginal 0.01% decline in the photo-generated current density is also attributed to applying a relatively weak electric field. The direct correlation between increased magnetic field strength and augmented photo-generated current density is an intriguing observation. For instance, under a fixed electric field condition, such as F = 0.4, the photo-generated current density ($j_{sc}$) increases from 76.216 to $76.229\;mA/cm^{2}$ as the magnetic field varies from 0 to 0.4, respectively, indicating a 0.2% augmentation. Therefore, this trend can be rationalized by the magnetic field’s substantial influence on the mobility and quantity of charge carriers traversing the junction, thereby enhancing the photocurrent. These results agree well with previous studies [30,31].

**Figure 6:**
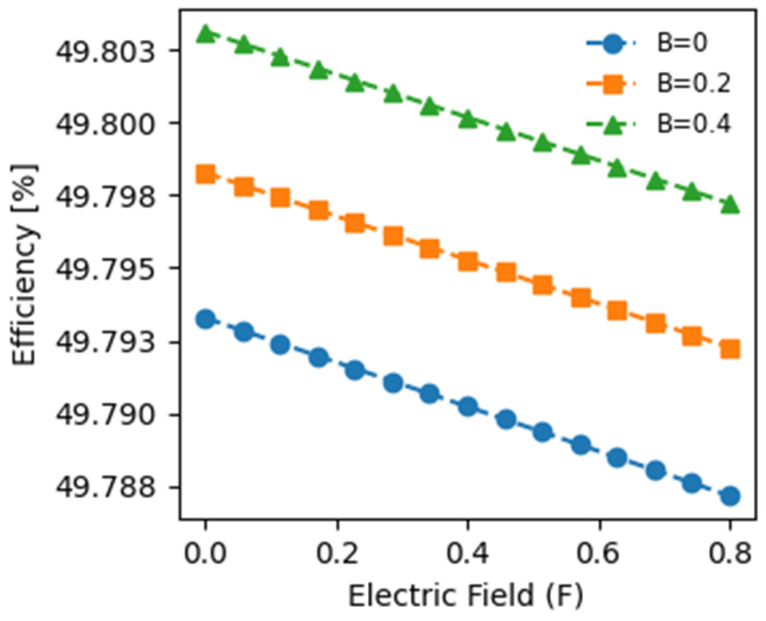
Variation in the performance of InGaN/GaN intermediate band solar cells (IBSCs) as a function of electric field intensity for three specified values of the applied magnetic field.

Figure 6 presents the photovoltaic conversion efficiency as a function of the electric field for three magnetic field values, considering the influences of impurities and heavy holes, which are typically neglected in similar studies. Our research underscores the significant promise of quantum well intermediate band solar cells (QW-IBSCs) under concentrated light, with efficiencies nearing 50%, marking a notable advancement in photovoltaic conversion. Furthermore, it has been demonstrated that maximum efficiency is attained at zero electric fields, irrespective of the magnetic field value, which aligns with the observed behavior for the open-circuit voltage and generated photocurrent density. Specifically, the maximum efficiencies achieved at F = 0 are 49.803%, 49.798%, and 49.793% for magnetic fields of 0.4, 0.2, and 0 T, respectively. This trend suggests that the optimal charge carrier dynamics occur when the electric field is minimized, likely reducing recombination losses and enhancing overall performance. Additionally, a slight decrease in efficiency is observed with increasing electric fields, regardless of the magnetic field value, with a minimal drop of around 0.01% as the electric field increases from 0 to 0.8. This behavior can be attributed to the Stark effect, where the presence of the electric field causes charge carriers to spread further apart. This increased spatial separation can negatively impact the $E_{12}$ and $E_{23}$ optical transitions, resulting in reduced photon absorption and decreased generated photocurrent density, ultimately diminishing efficiency. Conversely, efficiency improves with an increasing magnetic field at a fixed electric field. For instance, at F = 0, as the magnetic field varies from 0 to 0.4 T, the efficiency increases from 49.790% to 49.800%, indicating a 0.02% improvement. This enhancement may be due to the influence of the magnetic field on the charge carrier mobility and distribution, which can enhance the collection of charge carriers and reduce recombination rates, leading to improved photovoltaic performance.

Our results are consistent with previously reported works on the impact of electric and magnetic fields on photovoltaic conversion efficiency. In contrast to our study, an experimental study by Serafettin Erel [32] demonstrates that both electric and magnetic fields can influence the performances of photovoltaic cells under specific conditions (particularly with laser irradiation); Hassan Fathabadi’s work shows that external AC electric fields have no effect, while AC magnetic fields significantly reduce the PV power output of a silicon-based solar cell [33]. This comparison highlights a key difference in how alternating and direct current fields impact PV systems. This behavior contrasts with related studies, which typically report more pronounced effects from spontaneous emission sources, particularly at the nanoscale, where specific quantum phenomena can emerge and significantly influence performance.

This result underscores the beneficial impact of the magnetic field on photovoltaic conversion, attributed to increased confinement, leading to enhanced absorption, higher generated photocurrent density, and improved efficiency. This efficiency has demonstrated good consistency with previously published works [34,35]. It is crucial to note that our calculations assume ideal conditions with a fill factor equal to 1 (FF = 1) and full light concentration, leading to relatively high efficiencies compared to actual yields. They also assume an ideal fill factor of 1 and no recombination losses when exploring the theoretical upper limits of quantum well intermediate band solar cells (QW-IBSCs). While these assumptions help to illustrate the maximum potential efficiency, we acknowledge that non-radiative recombination is inevitable in practical devices, which would lower the real-world performance. Additionally, results for electric and magnetic fields in inclined directions are lower. Further studies will explore the effects of recombination, the light concentration factor, and the form factor on photovoltaic conversion efficiency.

## 4. Conclusions

This study investigated a novel intermediate band solar cell integrated within a standard p-i-n structure, operating at ambient temperature. Utilizing the finite difference method, we explored the potential of this cell to harness low-energy photons for electron transfer, salvaging otherwise wasted energy. Our computational analysis considered total light concentration, impurities, and heavy holes under perpendicular electric and magnetic fields. Our findings indicate that adjustments to both electric and magnetic fields can enhance energy conversion efficiency. However, the highest photovoltaic conversion efficiency of roughly 49.80% was achieved under a zero electric field and a magnetic field of B = 0.4, highlighting the role of magnetic fields in increasing current density through improved photon absorption. We believe that our work will contribute to advancing solar cell performance and may inspire further theoretical and experimental exploration in semiconductor materials.

## Figures and Tables

**Table 1:** The physical parameters used to perform the present numerical calculations [15,29].

Parameters	GaN	InN
$m_{e}^{*}/m_{0}^{*}$	0.19	0.10
$m_{h}^{*}/m_{0}^{*}$	0.81	0.83
$E_{g}\left(0\right)\;(\mathrm{eV})$	3.4	0.7
$\varepsilon^{*}$	8.68	11.6
$N_{V}\;({\mathrm{cm}}^{-3})$	$8\times 10^{15}\;T^{3/2}$	$10^{16}\;T^{3/2}$
$N_{C}\;({\mathrm{cm}}^{-3})$	$2.3\times 10^{14}\;T^{3/2}$	$1.76\times 10^{14}\;T^{3/2}$

## Data Availability

This will be available upon request. The original contributions presented in the study are included in the article, further inquiries can be directed to the corresponding author/s.
